# Comparative effectiveness of educational interventions in neurological disease for healthcare workers and students: a systematic review

**DOI:** 10.1136/bmjopen-2025-107475

**Published:** 2025-11-27

**Authors:** Munashe Veremu, Zhilin Jiang, Conor S Gillespie, Elena Roman, William H Cook, Rohil V Chauhan, Amir Rafati Fard, Georgios Toumbas, Shehla Baig, Carl Zipser, Sybil Stacpoole, Lindsay Tetreault, Naomi Deakin, Antony Bateman, Benjamin M Davies

**Affiliations:** 1 Division of Neurosurgery, Department of Clinical Neurosciences, University of Cambridge, Cambridge, UK; 2 National Hospital for Neurology and Neurosurgery, London, UK; 3 Active Living and Rehabilitation: Aotearoa New Zealand, Health and Rehabilitation Research Institute, Faculty of Health and Environmental Sciences, Auckland University of Technology, Auckland, New Zealand; 4 City St George’s University of London, London, UK; 5 Spinal Cord Injury Center and Department of Neurology and Neurophysiology, Balgrist University Hospital, University of Zurich, Zürich, Switzerland; 6 Neurology Unit, Department of Clinical Neurosciences, University of Cambridge, Cambridge, UK; 7 Department of Neurology, Massachusetts General Hospital, Boston, Massachusetts, USA; 8 Royal Derby Spine Centre, Royal Derby Hospital, Derby, UK

**Keywords:** Neurosurgery, Neurology, MEDICAL EDUCATION & TRAINING, Health Education

## Abstract

**Abstract:**

**Objectives:**

To assess the comparative effectiveness of educational interventions in neurological disease for healthcare workers and students.

**Design:**

Systematic review.

**Data sources:**

Medline, Embase and Cochrane through to 1 June 2025.

**Eligibility criteria:**

Studies evaluating neurological disease educational interventions with a comparator group (observational cohort/randomised controlled trial (RCT)) were included.

**Data extraction and synthesis:**

A Preferred Reporting Items for Systematic Reviews and Meta-Analyses-compliant systematic review was conducted (PROSPERO: CRD42023461838). Knowledge acquisition and educational methodologies were collected from each study. Study outcomes were classified using the Kirkpatrick and Kirkpatrick four-level model (learner reaction, knowledge acquisition, behavioural change, clinical outcome).^1^ Risk of bias was assessed using the Newcastle-Ottawa scale for non-randomised studies and the Cochrane Risk of Bias tool for RCTs.^2 3^

**Results:**

A total of 67 studies involving 4728 participants were included. Of these, 36 were RCTs, and 31 were observational studies. Virtual interventions were the most common (67.2%, n=45 studies), primarily targeting either medical students (46.3%, n=31 studies) or specialists (40.3%, n=27 studies). Overall, 70.1% (n=47) of studies demonstrated outcomes in favour of the intervention. However, few studies used K&K level 3/4 outcomes, with two studies evaluating behaviour change (level 3) and three assessing clinical outcomes (level 4 combined with other levels). No study exclusively assessed level 4 outcomes. Meta-analysis of 22 RCTs with calculable standardised mean differences (SMDs) (n=1748) showed a significant benefit of interventions (SMD 0.75, 95% CI 0.22 to 1.27, p=0.0056).

**Conclusions:**

This review highlights a growing body of research particularly focusing on virtual techniques, specialist audiences and treatment-oriented content. Few studies assessed changes in practice or patient care. Non-specialists remain underrepresented. Future studies should prioritise assessing the clinical impact of educational interventions within non-specialist audiences.

**STRENGTHS AND LIMITATIONS OF THIS STUDY**

- Identifies a large list of educational interventions for neurological disease which are focused on virtual interventions focussing on treatment on the audience of students.
- Identifies very few interventions targeting behavioural change or clinical outcomes.
- Lack of a common outcome measure and heterogeneity makes aggregation of outcomes challenging.

## Introduction

 Patient outcomes in neurological disease are affected by healthcare professional knowledge.^1^ Lack of knowledge may affect patient care through different mechanisms; however, it can also be a cause of delayed diagnosis.^2^,^4^ Neurophobia is widely recognised as the fear and difficulty in understanding neurological diseases and often results in poor knowledge retention and reluctance to engage with the field.^5^ This has far-reaching consequences, particularly in workforce planning and consequent clinical outcomes. A recent systematic review and meta-analysis on the prevalence of neurophobia revealed nearly half of medical students and young doctors included in their study having neurophobia.^6^ Another recent systematic review and meta-analysis found that students with neurophobia were 68% less likely to consider a career in neurology, with contributing factors including perceived complexity, limited exposure, the challenges of neurological examination and the emotional burden of neurology patients.^5^ Beyond discouraging neurology careers, neurophobia may persist among future general practitioners (GPs) and emergency physicians, compromising confidence in managing neurological presentations, which may contribute to delayed diagnosis. The complexity and diversity in clinical presentations of neurological conditions highlight the need for targeted, innovative educational strategies. While healthcare education is recognised as a priority, it is unclear how this can most effectively be improved.

Non-stroke neurological conditions account for 1 in 10 presentations to GPs and emergency departments,^2^ where non-specialist practitioners are often the first point of contact. These professionals need a broad understanding of clinically relevant facts to determine which cases require referral to overstretched specialist services and which can be managed in the community. A UK report found that over 40% of patients with neurological pathology saw a non-specialist at least five times before specialist referral.^3^ This delay is burdensome for indolent diseases and critical for progressive conditions, where delayed diagnosis adversely affects treatment outcomes. To support this, the UK National Institute for Health and Care Excellence commissioned a guideline for suspected neurological disease,^4^ attempting to help non-specialists navigate this complex challenge. While this has faced criticism, for example, with its scope and usability, it exemplifies the need for such education, with 84% of GPs in a UK national survey reporting a desire for further training to identify and manage patients presenting with neurological pathology.^7^

An illustrative case is degenerative cervical myelopathy (DCM), a common spinal pathology affecting approximately 1 in 50 adults, which initially presents with pain, sensorimotor and/or autonomic disturbances which can progress to paralysis.^8 9^ DCM is treatable through timely surgical intervention, but delayed diagnosis remains a significant issue. The literature shows that earlier rather than later intervention can be a positive predictor of recovery^10^; however, approximately 80% of patients present too late to gain maximal benefit from surgery.^11^ A recent survey found that nearly two-thirds of surveyed UK GPs were unfamiliar with the condition.^12^ A delayed diagnosis is common in progressive neurological conditions such as amyotrophic lateral sclerosis (ALS) and multiple sclerosis (MS).^13 14^ In ALS, diagnostic delay is frequently attributed to limited recognition among primary care ‘gatekeepers’, with improved education suggested as a key intervention.^14^ Similarly, studies in MS highlight knowledge gaps at non-neurological consultations as contributors to delay,^13^ emphasising the need for greater awareness across the diagnostic pathway, particularly within non-specialist cohorts who have shown willingness to learn about neurological pathology.^15^

This systematic review summarises primary research evaluating educational interventions in neurological disease pertaining to both patients requiring either medical or surgical management. Virtual interventions, including simulation and e-learning, have emerged as promising solutions, though their real-world impact on clinical outcomes remains uncertain. The most relevant previous systematic review was published more than a decade ago and identified a limited number of andomised controlled trials (RCTs) in neurological education. It excluded neurosurgical studies and focused on basic knowledge acquisition rather than clinical application.^16^

This review examines the comparative effectiveness, educational efficiency and success of educational interventions in neurological disease targeting healthcare professionals and students. This review also explores the types of interventions evaluated, the target population/s and the methods used for intervention assessment. The inclusion of patient outcomes (eg, procedural complications following a procedural educational intervention) and the extent to which the presented interventions led to improvements in clinical practice are critically assessed with the use of the Kirkpatrick and Kirkpatrick (K&K) evaluation model.^17^ The review also investigates how studies defined and measured the success of their educational interventions. The review identifies gaps in the current literature and sets out key priorities for future research in educational approaches within neurological disease management.

## Methods

### Search strategy and selection criteria

We conducted a systematic review following the Preferred Reporting Items for Systematic Reviews and Meta-Analyses guidelines.^18^ The review was prospectively registered in PROSPERO (CRD42023461838); the protocol remained unchanged prior to manuscript writing.

We searched Medline, Embase and CENTRAL (Cochrane) for full-text articles published in English from inception to 8 September 2023 (updated 1 June 2025).^19^,^21^ Only English studies were included due to high numbers of papers expected and feasibility of translation among the authorship team. The search terms were limited to combinations of the following keywords: “educational intervention”, “neurology”, “neurosurgery”, “neuroscience”, plus all synonyms, abbreviations and variations in spellings of these terms, using Boolean operators (online supplemental figures 1 and 2).

The Population, Intervention, Comparator, Outcome, Study design criteria were applied (table 1).^22^ We included studies of adults (≥18 years old) who are healthcare students/professionals learning about a neurological/neurosurgical disease. Studies were excluded if they included participants learning about non-neurological disease, the population were non-healthcare practitioners or the general public or the study did not include an evaluation of the educational intervention.

**Table 1:** Population, Intervention, Comparator, Outcome, Study design (PICOS) criteria

	Inclusion	Exclusion
Review question	To assess the comparative effectiveness of educational interventions in neurological disease for healthcare workers and students.	
Population	Adults≥18 years who are healthcare students/professionals learning about neurological/neurosurgical disease. These studies took place in any university, neurosurgery, trauma or radiology/neurology department or centre.	Participants learning about non-neurological disease. The population was non-healthcare practitioners or the general public.
Intervention	Any educational intervention (eg simulation/ virtual reality) used with the purpose of improving education of a neurological/neurosurgical disease(s).	
Comparator	Any comparator/control group against the educational intervention.	Studies without a comparator group.
Outcomes	Primary: Identify the effectiveness of the educational intervention. Identify Kirkpatrick and Kirkpatrick evaluation level of the intervention. Secondary: Identify the methodologies used (including target audience, method of intervention and educational objective).	
Study design	Must be a randomised trial or an observational cohort study with an intervention and control/comparator group. Single-arm studies were excluded.	The study did not include an evaluation of the educational intervention.

Five reviewers (MV, ZJ, ER, CSG, GT) independently screened the titles, abstracts and full texts to select the articles for inclusion. Manual study deduplication and initial screening of titles/abstracts were conducted using Rayyan (Rayyan Systems Inc, Cambridge, MA, USA). Where consensus could not be reached, a senior author (BD/LT) was consulted to assist with making the final decision. We identified and manually excluded duplicates and consolidated multiple reports of the same study to ensure that each study, rather than each individual report, served as the unit of analysis for the review. Additional eligible studies identified during peer review were incorporated post hoc if they satisfied the predefined eligibility criteria.

### Data extraction

Data extraction was completed independently by two authors for each study. For each included study, we gathered data on the year of publication, journal, study design (RCT or observational), population characteristics, geographic location, disease area (classified as the brain, spinal cord or peripheral nervous system), method of intervention (virtual or non-virtual in delivery) and the evaluation of intervention effectiveness mapped to the K&K model (identifying the highest level reached: level 1, reaction; level 2, learning; level 3, behavioural change; level 4, clinical results). Educational objectives were recorded as diagnosis, examination or treatment. The target audience was noted as students, specialists, non-specialists or allied health professionals (AHPs).

### Quality and certainty assessment

Observational studies were assessed according to the Newcastle Ottawa scale and RCTs according to the Cochrane Risk of Bias 2.0 tool.^23 24^ The certainty of the body of evidence was evaluated using the Grading of Recommendations, Assessment, Development and Evaluations (GRADE) framework. Two authors (MV, CG) independently conducted quality and GRADE assessments, resolving any disagreements through discussion.^25^

### Definitions

In this context, a neurological disease was defined as any pathology, of any aetiology, that affects the brain, spine or peripheral nervous system either independently or simultaneously. An educational intervention was defined as any teaching innovation, simulation or event that aims to transfer knowledge to a population. The participants were expected either to be present at the teaching intervention or selected to participate in the teaching intervention at their convenience within a set timeframe. The population of interest was adult healthcare professionals or students studying to become a healthcare professional (>18 years old) who are learning about neurological disease. Healthcare professionals may include (not exhaustive) physiotherapists, nurse practitioners, occupational therapists, chiropractors, osteopaths, physician associates, nurses and doctors. A virtual intervention was defined as a non-literal and immersive experience delivered in person on a computer/simulator or via an online virtual platform. Specialists were either recognised specialty residents/trainees or consultant/attendings within neurological disease. An intervention was deemed favourable or effective if the intervention caused an improvement (any positive trend) in the primary outcome. All studies were analysed on their use of the K&K model of evaluating an intervention and categorised into four levels: level 1 (learning reaction), level 2 (learning assessment), level 3 (behavioural change) and level 4 (change in clinical outcome).

### Statistical analysis and data synthesis

All results were presented using descriptive statistics including frequencies, percentages, means, median, SD, range and IQR. For each study, we calculated the standardised mean difference (SMD) between intervention and control groups (where the SMD was calculable). Where multiple outcomes were reported for the same study, we selected the most relevant outcome for our review question (overall knowledge/skills at post-test). Other outcomes are presented narratively. Hedges’ g was used to correct for small sample bias. Pooled SMDs and 95% CIs were estimated using a random-effects model with inverse-variance weighting to account for methodological and clinical heterogeneity across studies. Statistical heterogeneity was assessed using Cochran’s Q test, the I² statistic and τ². Forest plots were generated to display study-level effect sizes and the pooled estimate. Some RCTs were excluded from SMD calculation where post-test means and SDs were not reported, outcomes were presented only as change scores, ranges or binary measures or analyses were conducted at a non-participant level. In other cases, only SEMs were available, which would have required back-calculation. All analyses and figure generation were carried out using R (RStudio V.4.0.1, R Core Project, Vienna, Austria; ggplot, tidyverse, metafor and meta packages), Microsoft Excel (V.16.90, Microsoft, Redmond, WA, USA) and jamovi V.2.5 (jamovi project, Sydney, Australia).^26^

## Results

### Systematic review and characteristics

**Figure 1:**
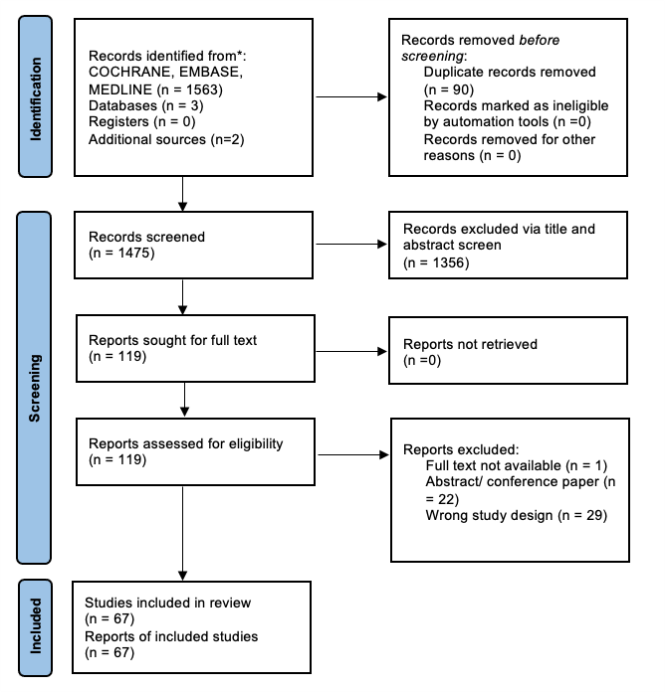
Preferred Reporting Items for Systematic Reviews and Meta-Analyses (PRISMA) flow chart.

Our search yielded a total of 1473 publications. Following title and abstract screening, the full texts of 117 studies were assessed for potential inclusion. An additional 50 studies were excluded (figure 1) following a full-text assessment. This yielded 67 studies for inclusion after applying the inclusion and exclusion criteria. Of these, 36 studies were RCTs, and 31 were observational studies. RCTs included a combined total of 2973 participants, with a median number of participants per study of 64 (IQR 35.0–120.0, range 10–417). Observational studies included a total of 2370 participants, with a median number of participants per study of 37.0 (IQR 17.5–64.0, range 6–360). 70.1% of the educational interventions were deemed ‘effective’.

### Baseline characteristics

The baseline characteristics are summarised in table 2. Geographically, 38.8% of the studies were conducted in the USA, with Canada and China comprising the remainder of the top 50%.

**Table 2:** Baseline characteristics of the study

Baseline characteristics	Value (%) (**IQR**)
Total number of studies included	67
Total number of participants in studies	5235
Median number of participants per study	52 (23.0–100.0)
**Studies with a power calculation**	18 (26.9)
**Study design**	**Frequency (%)**
Observational	31 (46.3)
RCT	36 (53.7)
**Journal**	**Frequency (%)**
World Neurosurgery	10 (14.9)
BMC Medical Education	9 (13.4)
Operative Neurosurgery	4 (6.0)
Anatomy Sciences Education	3 (4.5)
Other	41 (60.0)
**Method of Intervention**	**Frequency (%)**
Virtual	45 (67.2)
Non-virtual	20 (29.9)
Both	2 (3.0)
**Audience**	**Frequency (%)**
Medical students	31 (46.3)
Specialists	27 (40.3)
Non-specialists	1 (1.5)
AHPs	3 (4.4)
Specialists and medical students	4 (6.0)
**Specialists and AHPs**	1 (1.5)
**Target of education**	**Frequency (%)**
Diagnosis	21 (31.3)
Examination	7 (10.4)
Treatment	31 (46.3)
Diagnosis and treatment	8 (11.9)
**Disease area**	**Frequency (%)**
Brain	38 (56.7)
Spinal cord	9 (13.4)
All	20 (29.9)
**Kirkpatrick level**	**Frequency (%)**
1 (learner reaction)	8 (11.9)
2 (assessment)	33 (49.3)
3 (behavioural change)	2 (3.0)
4 (clinical results)	0 (0)
Combination (1 and 2 or 1, 2 and 3 or 1 and 4 or 2 and 3)	24 (35.8)
**Effective**	**Frequency (%)**
Yes	47 (70.1)
No	20 (29.9)

AHPs, allied health professionals (eg, physiotherapists); RCT, randomised controlled trial.

### Evaluation of educational interventions

**Figure 2:**
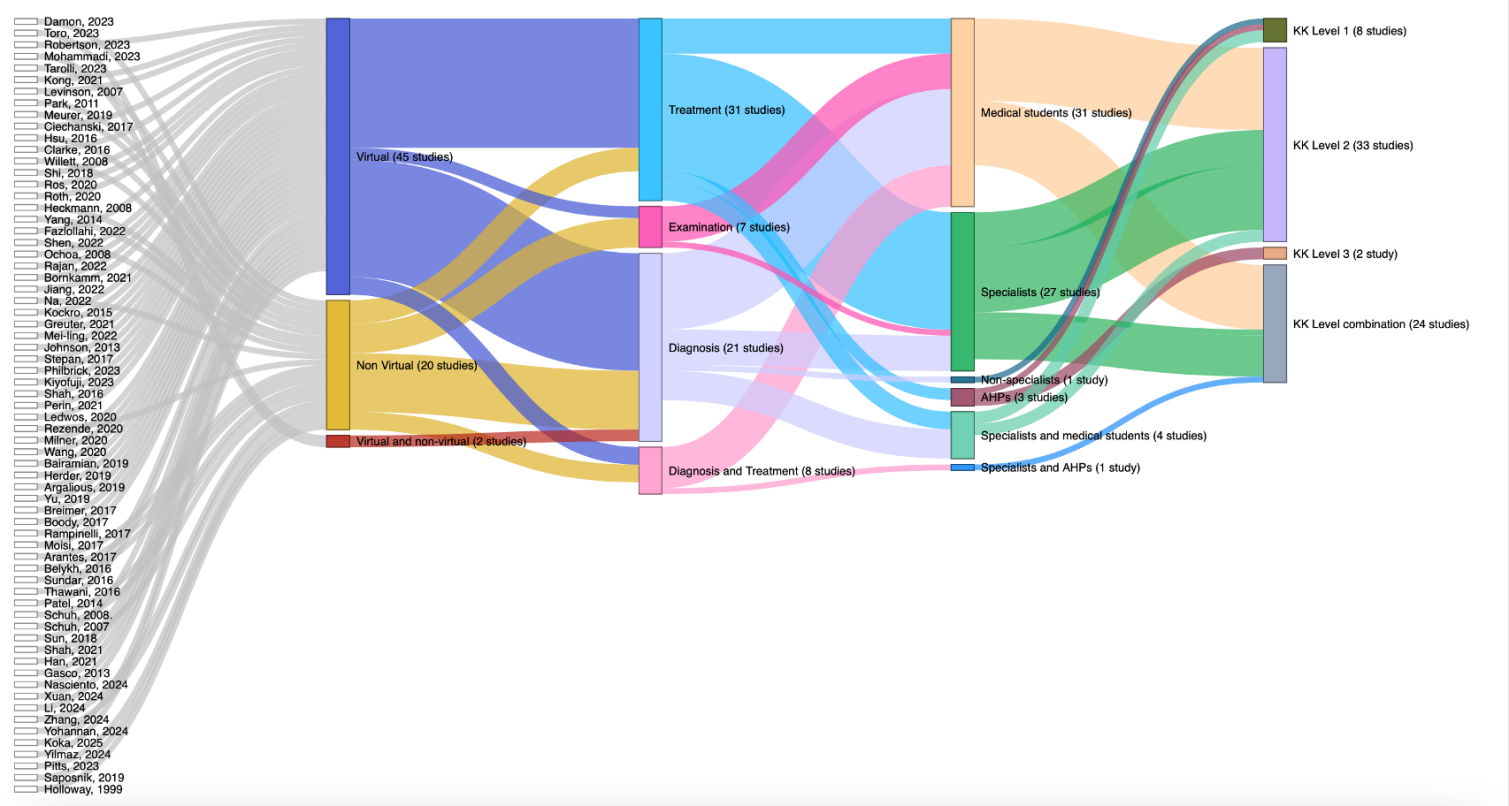
Alluvial plot showing the study characteristics of all 67 studies included in the analysis. AHPs, allied health professionals; KK, Kirkpatrick & Kirkpatrick.

Based on the K&K model for evaluating educational interventions, 11.9% (n=8) of interventions used a learning assessment (level 1) to evaluate their educational intervention, while 49.3% (n=33) of studies used learners’ reaction (level 2). Two (3.0%) studies evaluated their study based on a behavioural change (level 3). Zero studies evaluated their study based principally on clinical results (level 4). Finally, 24 (35.8%) studies used a combination of the K&K models to evaluate their study (eg, learning reaction and learning assessment). These findings are summarised in figure 2.

Node and link size are proportional to the number of studies. Study classification is by methodology (virtual, non-virtual or mixed), focus (treatment, examination, diagnosis or combined) and target audience (medical students, specialists, allied professionals or non-medical individuals). Studies are mapped to K&K levels 1–3 or a combination of levels demonstrating their alignment with this evaluation framework.

### Studies which evaluated their educational interventions by learning reaction (Kirkpatrick and Kirkpatrick level 1)

In total, eight studies solely evaluated their educational intervention using learning reaction (online supplemental table 1). All studies were observational, employed virtual interventions (mainly comparing standard practice with simulated environments such as virtual reality [VR], 3-dimentional [3D] models or cadaveric/animal simulations for surgical training as one time point events) and focused on treatment. All targeted specialists, with two also including medical students.^27 28^ Gasco *et al* used medical students to determine if this educational intervention also develops career interest.^28^ Three studies were aimed at increasing knowledge of the treatment of spinal cord pathology,^29^,^31^ whereas the remaining five were aimed at treating brain pathology.^2728 32^,^34^

### Studies evaluated by learning assessment (Kirkpatrick and Kirkpatrick level 2)

A total of 33 studies exclusively evaluated their educational intervention using learning assessment (online supplemental table 2).^35^,^57^ Of these, 61% (n=20) were RCTs, while the remaining were observational studies. Virtual interventions were used in 67% (n=22) of the learning assessment studies.

Fifteen studies (45%) focused on diagnosis, and 13 (39%) studies were aimed at treatment. A single study addressed both diagnosis and treatment. The remaining studies were centred on examination.

Most studies (52%, n=17) were solely directed at medical students, while 33% (n=11) targeted specialists. Two studies encompassed both target populations, and one study each was aimed at AHPs and non-specialists.

Two studies specifically addressed spinal cord pathology.^58 59^ Notably, both were RCTs and the intervention arms incorporated simulation. Additionally, nine studies encompassed all disease areas (spinal cord, brain and peripheral nervous system), whereas the remaining 66% of studies (n=22) focused on brain pathology.

Across the 33 studies, 66% assessed learning with pre/post written tests, and just 15% used validated or externally standardised instruments such as the Residency In Service Examination^60^; the remainder relied on bespoke quizzes or did not report validation. Zero studies used real patients.

### Studies evaluated by behavioural change (Kirkpatrick and Kirkpatrick level 3)

Two studies^61 62^ (online supplemental table 3) evaluated a behavioural change following an educational intervention. One is an observational study which used a virtual intervention combining simulation and cadaveric specimens to educate specialists in the treatment of spinal cord pathology. The study intervention group showed fewer screw placement errors in a laboratory setting when compared with the control group (didactic teaching alone).^61^ The second study is an RCT which investigated the use of a reflective educational intervention (traffic light system, TLS) on therapeutic inertia in simulated MS cases among specialist neurologists. The TLS group (intervention group) showed a relative reduction of therapeutic inertia of approximately 70% when compared with the control group, demonstrating a change in behaviour.^62^

### Studies evaluated by change in clinical outcome (Kirkpatrick and Kirkpatrick level 4) and studies which used a combination of Kirkpatrick and Kirkpatrick levels

No studies were exclusively assessed for a change in clinical outcome following an educational intervention. Only three studies included an evaluation of clinical outcome in conjunction with learning reaction.^63^,^65^ One RCT in dementia care evaluated a multifaceted educational intervention comprising an education course, practice-based tools, an expert-led seminar and mailed materials.^65^ Outcomes were assessed through clinician surveys at 3-month intervals and review of patient records. The intervention group demonstrated greater adherence to at least three of six guideline recommendations compared with the control group.

A total of 24 studies (35.8% of all included studies) combined K&K models for evaluation (online supplemental table 3).^66^,^81^ The most common combination was learner reaction (K&K 1) and learner assessment (K&K 2), accounting for 79.2% of studies. Nearly an equal proportion of the studies were observational studies (60% observational vs 55% RCT). Regarding the use of technology, 62.5% (n=15) of studies used only virtual educational interventions, while the remaining employed either non-virtual or combined virtual and non-virtual approaches.

Ten studies were centred on treatment, seven on diagnosis, with five studies addressing both diagnosis and treatment. An additional two studies were focused on examination. Most (54.2%, n=13) targeted medical students, with 29.2% (n=7) designed for specialists. One study encompassed both populations, two targeted AHPs and the remaining study targeted students and AHPs. Three studies specifically addressed spinal cord pathology,^63 82 83^ whereas the remainder were either focused on intracranial pathology or encompassed all neurological pathology (brain, spinal cord, peripheral nervous system).

### Comparative effectiveness and standardised mean difference

**Figure 3:**
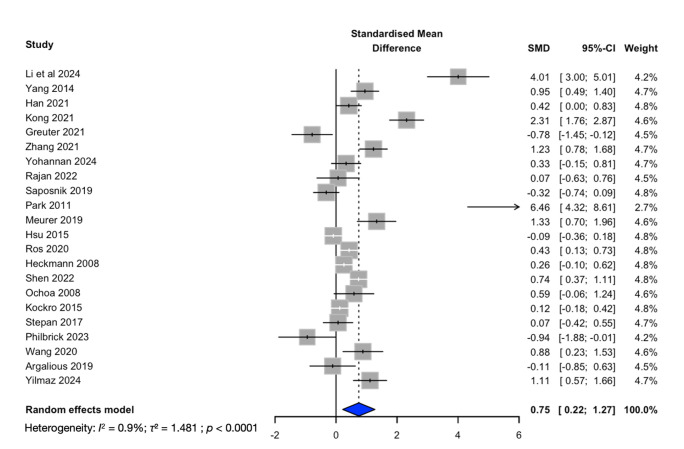
Forest plot for the standardise mean difference for intervention versus control. Comparing intervention versus control across 22 randomised controlled trials (RCTs) (n=1748). Pooled analysis using a random-effects model favoured the intervention (standardised mean difference (SMD) 0.75, 95% CI 0.22 to 1.27). SMD, standardised mean difference.

A summary of the comparative effectiveness of educational interventions in neurological disease is summarised in table 3, with the comparative effectiveness by disease area, target of education, method of education and target audience. The certainty of evidence (table 3) was rated as moderate or low across all categories, primarily due to the predominance of non-randomised study designs and, in the case of randomised studies, an increased risk of bias. Additionally, inconsistency across studies further contributed to the downgrading of evidence certainty. Twenty-two RCTs (n=1748) were included in the analysis for the SMD. The pooled effect demonstrated a significant benefit of interventions compared with controls (SMD 0.75, 95% CI 0.22 to 1.27, p=0.0056) (figure 3).

**Table 3:** Summary of comparative effectiveness of educational interventions and certainty of evidence using the Grading of Recommendations, Assessment, Development and Evaluations (GRADE) framework split by disease area, target of education, method of education and target audience

Category	Number of studies (%)	Number of effective studies	Effectiveness rate (%)	Certainty of evidence (GRADE)
Disease area				
Brain pathology	38 (56.7)	28 (18 RCTs; 10 NRSIs)	73.7	⨁⨁◯◯ Low Due to risk of bias and inconsistency
Spinal cord disease	9 (13.4)	8 (1 RCT; 7 NRSIs)	88.8	⨁⨁◯◯ Low Due to risk of bias and inconsistency
All	20 (29.9)	11 (6 RCTs; 5 NRSIs)	55.0	⨁⨁◯◯ Low Due to risk of bias and inconsistency
Target of education				
Treatment	31 (46.3)	24 (9 RCTs; 15 NRSIs)	77.4	⨁⨁◯◯ Low Due to risk of bias and inconsistency
Diagnosis	21 (31.3)	14 (11 RCTs; 3 NRSIs)	66.7	⨁⨁◯◯ Low Due to risk of bias and inconsistency
Diagnosis and treatment	8 (11.9)	5 (4 RCTs; 1 NRSI)	62.5	⨁⨁◯◯ Low Due to risk of bias and inconsistency
Examination	7 (10.4)	4 (1 RCT; 3 NRSIs)	57.1	⨁⨁◯◯ Low Due to risk of bias and inconsistency
Method of education				
Virtual	45 (67.2)	33 (16 RCTs; 17 NRSIs)	75.0	⨁⨁◯◯ Low Due to risk of bias and inconsistency
Non-virtual	20 (29.9)	14 (8 RCTs; 6 NRSIs)	70.0	⨁⨁◯◯ Low Due to risk of bias and inconsistency
Both virtual and non-virtual	2 (3.0)	0	0.0	⨁⨁◯◯ Low Due to risk of bias and inconsistency
Target audience				
Medical students	31 (46.3)	19 (15 RCTs; 4 NRSIs)	61.3	⨁⨁◯◯ Low Due to risk of bias and inconsistency
Specialists	27 (40.3)	21 (8 RCTs; 13 NRSIs)	77.8	⨁⨁◯◯ Low Due to risk of bias and inconsistency
Specialists and medical students	4 (6.0)	4 (1 RCT; 3 NRSIs)	100.0	⨁⨁◯◯ Low Due to risk of bias and inconsistency
Non-specialists	1 (1.5)	1 (1 NRSI)	100.0	⨁⨁◯◯ Low Due to risk of bias and inconsistency
AHPs	3 (4.5)	1 (1 NRSI)	33.3	⨁⨁◯◯ Low Due to risk of bias
Specialists and AHPs	1 (1.5)	1 (1RCT)	100	⨁⨁⨁◯ Moderate Due to risk of bias
Kirkpatrick and Kirkpatrick level				
K&K level 1	8 (11.9)	6 (6 NRSIs)	75.0	⨁⨁◯◯ Low Due to risk of bias and inconsistency
K&K level 2	33 (49.3)	26 (16 RCTs, 10 NRSIs)	78.8	⨁⨁◯◯ Low Due to risk of bias and inconsistency
K&K level 3	2 (3.0)	2 (1 RCT, 1 NRSI)	100.0	⨁⨁◯◯ Low Due to risk of bias
K&K level combo	24 (35.8)	13 (8 RCTs, 5 NRSIs)	54.2	⨁⨁◯◯ Low Due to risk of bias and inconsistency

AHPs, allied health professionals; GRADE, Grading of Recommendations, Assessment, Development and Evaluations; K&K, Kirkpatrick and Kirkpatrick; NRSI, non-randomised study of interventions; RCTs, randomised controlled trials.

**Figure 4:**
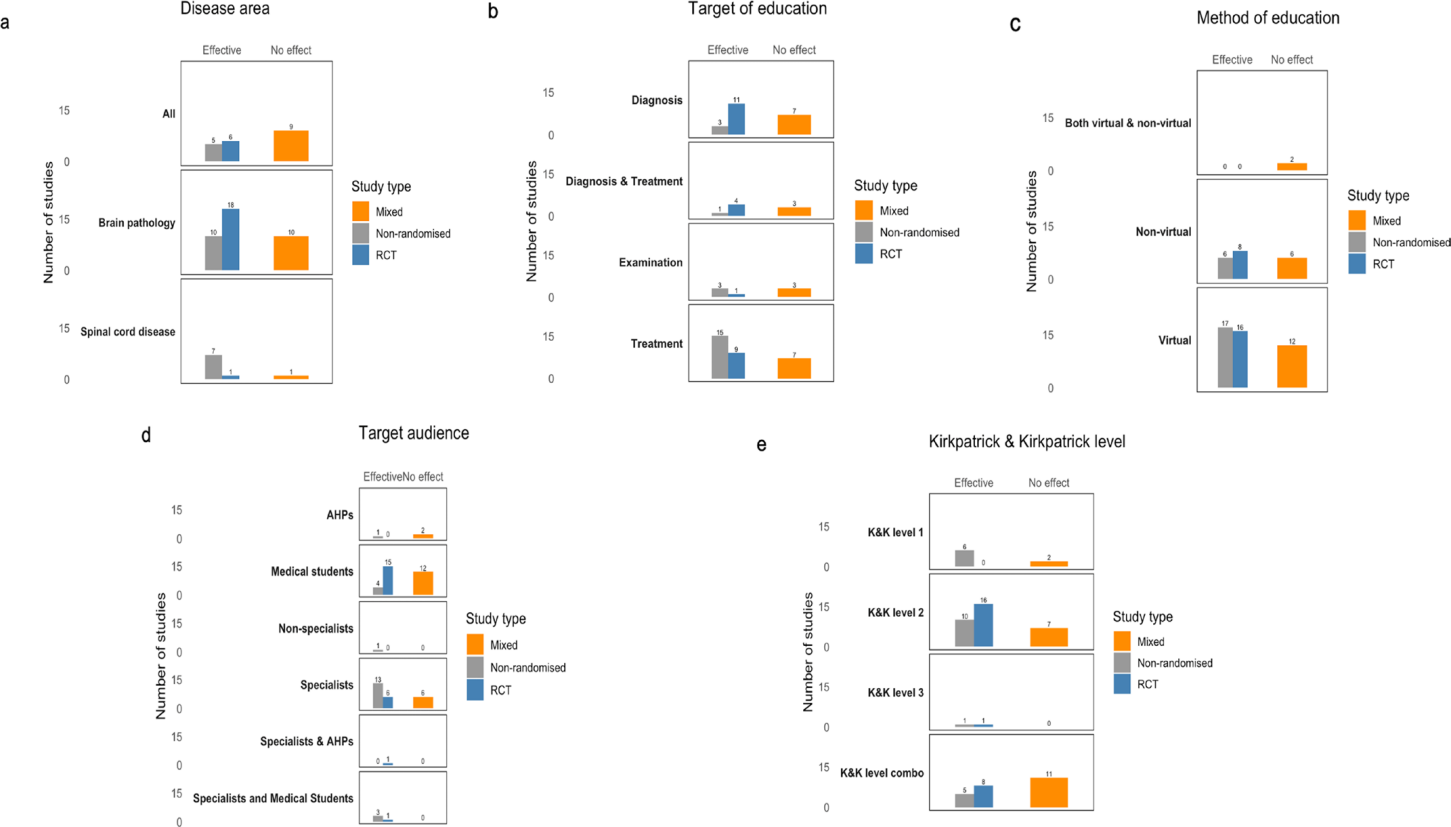
Harvest plot of (a) disease area, (**b**) target of education, (**c**) method of education, (**d**) target audience and (**e**) Kirkpatrick and Kirkpatrick level. Bar height represents number of studies, with colours distinguishing randomised controlled trials (RCTs), non-randomised study of interventions (NRSIs), with the no effect row having a mix of both RCTs and non-randomised studies. AHP, allied health professionals; K&K, Kirkpatrick and Kirkpatrick; RCT, randomised controlled trial.

The differences in the intervention outcomes across all the categories split by disease area, target of education, method of education, K&K level and target audience are further shown in figure 4. Overall, the evidence was mixed across all subcategories, with both RCTs and NRSIs showing variable effectiveness. Within each category—disease area (figure 4a), target of education (figure 4b), method of education (figure 4c), target audience (figure 4d) and K&K level (figure 4e)—a similar pattern emerged, with many studies reporting a benefit; however, some still reported no effect.

### Assessment of bias

Observational non-randomised studies were assessed according to the Newcastle Ottawa scale and RCTs according to the Cochrane Risk of Bias 2.0 tool (online supplemental table 4 and figure 3). Nearly half of all included studies were non-randomised and therefore at risk of bias, including increased risk of unmeasured confounding variables and selection bias. The mean score on the Newcastle Ottawa scale (out of 9) for the included studies was 5.91, with 23 studies classed as high risk of bias (due to compatibility). For the RCTs, 5 were categorised as low risk, 2 were at high risk and 29 studies were categorised as moderate risk (online supplemental figure 3). The RCTs used for the SMD are displayed in a funnel plot (online supplemental figure 4).

## Discussion

### Key findings

This systematic review comprehensively examines the primary literature, highlighting interventions aimed at educating healthcare professionals and students about neurological conditions. The findings indicate an increase in primary research on neurological educational interventions over time, with a notable recent emphasis on virtual teaching methodologies, targeting specialist audiences with treatment-focused content. While most studies report favourable outcomes, only a small proportion assessed outcomes based on practice changes or improvements in patient care. Despite the widespread recognition of the importance of enhancing neurological knowledge to reduce diagnostic delays, the optimal approach for achieving this in a scalable and cost-effective manner therefore remains unclear. Further, the transferability of the findings to non-specialist professionals is unclear, and interventions to this group remain critically under-researched.

### Increased volume of primary research

The volume of primary research evaluating neurological educational interventions has increased, with a particular emphasis on virtual methodologies, specialist audiences and treatment-focused approaches. A prior systematic review conducted in 2013 highlighted a lack of high-quality evidence supporting educational interventions in neurological education, particularly excluding neurosurgical studies, with only 16 RCTs.^16^ In contrast, as illustrated in table 2, our updated review reflects a significant increase in both randomised and non-randomised educational interventions in recent years. Even after considering slight variations in search criteria between the two reviews, our analysis identified 36 RCTs, accompanied by a substantial number of non-randomised studies. This observed increase reflects sustained efforts to enhance neurological education.

### Predominance of virtual interventions

Virtual interventions were the most common approach, with nearly two-thirds of included studies using virtual interventions. This reflects the findings of a recent scoping review of innovations in clinical neurology education,^84^ which reported that most included studies used simulation or e-learning. Despite the exclusion of neurosurgical studies, their pattern aligns with that of our review. The effect of the COVID-19 pandemic and the resultant surge in virtual educational events must be acknowledged as a precipitant.

All studies using both virtual and non-virtual methods showed no significant differences between groups.^33^ This might be explained by the meshing hypothesis,^85^ which suggests that learners perform better when the teaching method aligns with their learning style (eg, visual learners with visual activities). Combining multiple teaching methods may lessen the impact of each, particularly if some do not suit a learner’s style, which could explain the lack of difference between groups. However, selecting participants for a particular intervention based on their learning styles may introduce other types of bias. There are further considerations to virtual methods which include the cost of developing the virtual intervention, coupled with the cost to the learner of accessing the virtual intervention. This is something which educators ought to consider when developing virtual interventions.

### Favourable outcomes

While most studies report a favourable outcome, some do not, and very few have used measures of change in practice (K&K level 3) or improved patient care (K&K level 4). Reaction-based evaluations were the most common outcome measure. They are arguably the simplest to replicate, as they often involve self-reported satisfaction surveys or assessments of participants’ opinions regarding the intervention. However, they may not provide an accurate measure of its effectiveness, and there are concerns about the reliability of learner satisfaction as an evaluation metric. Evidence shows that participants are often poor judges of their own competence within a given subject area^86 87^ and that post-intervention satisfaction can be misleading.^88^ For instance, recent findings indicate that participants engaged in active learning strategies are more likely to report dissatisfaction, despite demonstrating higher levels of achievement in their assessments.^89^ This highlights the need for more objective assessment methods.

Only a small number of studies evaluated interventions at Kirkpatrick level 4, and none used this as a primary outcome. Examples included studies reporting surgical complication rates following an educational intervention, such as dural leaks, nerve injuries or post-operative visual impairment.^63 63 64 64^ This trend aligns with findings from previous reviews on neurological education: specifically, the aforementioned systematic review from 2013 identified only two studies employing K&K level 4 outcomes for their educational interventions,^16^ and a subsequent scoping review on innovations in clinical neurology reported three additional studies using these higher-level outcomes.^84^

### Diagnostic interventions

Addressing diagnostic delays in neurological conditions in a scalable and cost-effective manner remains challenging. Our review identified 21 studies focusing on diagnostic interventions, of which 14 were deemed effective. Of the effective interventions, 78.5% (n=11) were evaluated by RCTs, with the majority targeting medical students and specialists using virtual platforms. One study focused on dizziness, a symptom associated with a myriad of conditions ranging from benign labyrinthine disorders to acute stroke—grossly highlighting the diagnostic challenges given the stark differences in morbidity and mortality between these pathologies. The intervention targeted benign paroxysmal positional vertigo by educating emergency physicians on the Dix-Hallpike test and canalith repositioning manoeuvre. The authors used an RCT methodology and demonstrated improved diagnostic accuracy among participants who received a combined clinical vignette and computer lecture intervention. This method was effective and would potentially be scalable for widespread implementation due to the relative ease of delivering a clinical vignette and lecture intervention to more clinicians.^45^ Future efforts should prioritise scalable, evidence-based strategies that can be integrated into clinical practice to enhance early diagnosis and improve patient outcomes.

### Limitations and future directions

This study has several limitations. The substantial heterogeneity in study design and reported outcomes prevented data aggregation and precluded conduction of a planned meta-analysis. The pooled effect demonstrated a significant benefit of interventions compared with controls. However, heterogeneity was high (I²=90.3%, τ²=1.48), indicating considerable variability across studies. Several eligible RCTs were excluded from pooled analysis because necessary summary statistics (means, SDs or equivalent) were not available for SMD calculation.

A key limitation of this review is the broad scope of various conditions and specialties, making it challenging to interpret favourable results consistently across studies. Additionally, there is significant variation in the types of educational interventions and clinical tasks analysed. Some interventions demand vastly different skillsets, from the inherently surgical-manual dexterity and spatial reasoning to cognitive knowledge of theoretical pathology and physiology. This heterogeneity complicates direct comparisons between the interventions and limits the generalisability of the findings across all included clinical scenarios. Further, the progressive development of newer, more advanced educational interventions over time may render earlier findings less relevant, and we cannot ascertain how educational technologies used in 2018 would perform with learners in 2025. Additionally, many of the included studies exhibited a high risk of bias, further reducing the generalisability of the results.

These findings highlight the ongoing scarcity of research evaluating the direct impact of educational interventions on patient care. To address this gap, future prospective longitudinal studies are necessary to more effectively assess the influence of educational interventions on clinical outcomes. Implementing K&K level 4 outcomes within studies may incur policy and curriculum implications, such as by integrating level 4 outcomes into accreditation standards for continuing professional development. A consistent lack of standardised outcome measures across studies further complicates the comparison of results. The development of a widely applicable, standardised educational assessment or retention tool would be beneficial for evaluating the effectiveness of future interventions and enhancing the comparability of future trials.

## Conclusions

In conclusion, this systematic review evaluates the available evidence for educational interventions in neurological diseases. The findings reflect a focus on virtual methods for specialist audiences, educating about the treatment of neurological pathology. Although many studies report positive outcomes, few assess changes in practice or patient care improvements, and for the purpose of accelerating diagnosis, few evaluate interventions for non-specialist professionals. Future studies should prioritise assessing the clinical impact of educational interventions within non-specialist audiences.

## Supplementary material

10.1136/bmjopen-2025-107475 online supplemental file 1

## Data Availability

Data are available upon reasonable request.
