# Supplementary material for: Comparative effectiveness of educational interventions in neurological disease for healthcare workers and students: a systematic review
Source: BMJ Open. 2025 Nov 27;15(11):e107475. doi: 10.1136/bmjopen-2025-107475 (PMC12666186; doi:10.1136/bmjopen-2025-107475)

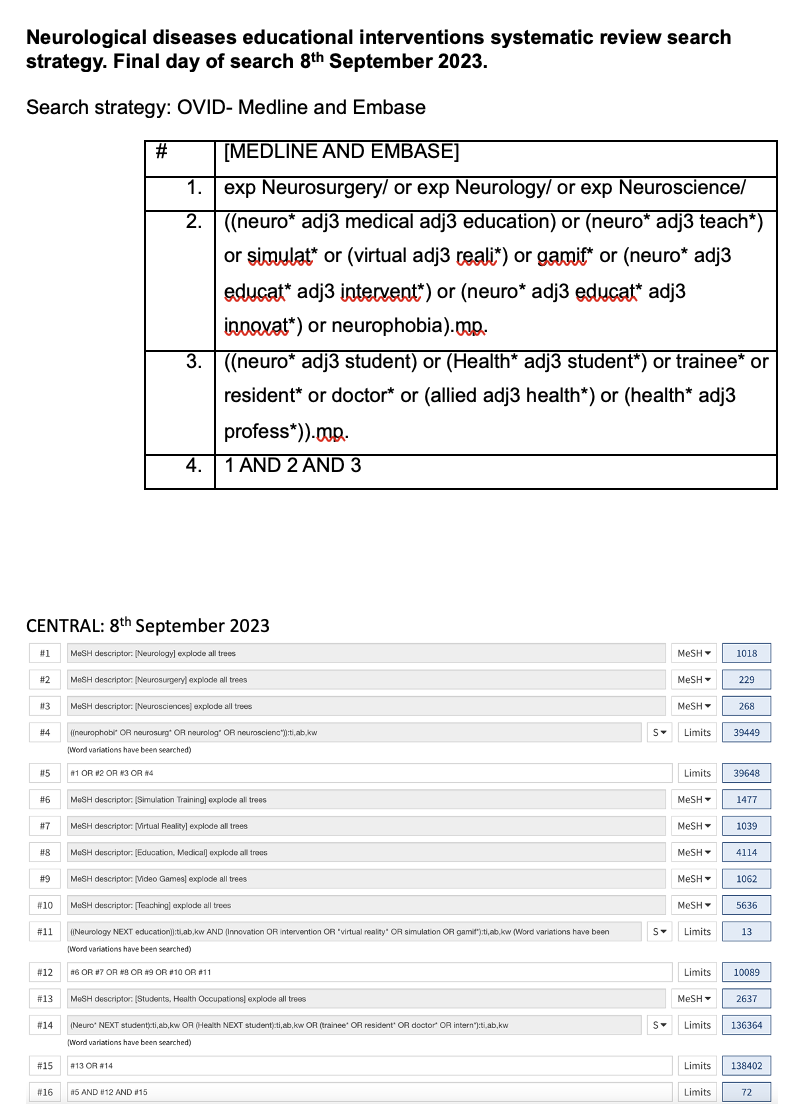


**Supplementary Figure 1** Search strategy


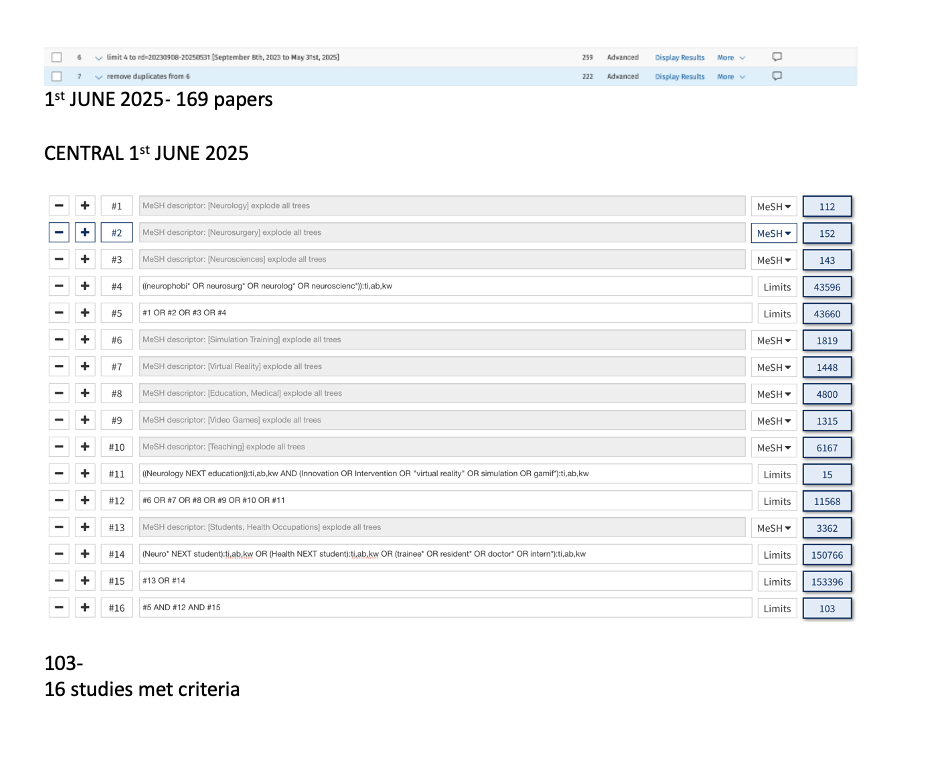


**Supplementary Figure 2.** Repeat Search results. 1st June 2025.

**Supplementary Table 1**. Studies exclusively assessing educational interventions by learning reaction (K&K Level 1)

| Study (Author/Year) | Disease Area | Intervention | Method  (virtual vs non virtual) | Type of study | Objective of intervention | Audience | Main outcome (s) | Findings |
| --- | --- | --- | --- | --- | --- | --- | --- | --- |
| Damon Et al. Oper Neurosurg 2023.[31] | Spinal Cord | Simulation vs cadaveric models | Virtual and  Non-virtual | Observational | Treatment | Specialist | Learner perception | High satisfaction with simulator. |
| Ledwos et al Oper Neurosurg 2020.[30], [34] | Spinal Cord | Virtual reality (VR) simulation | Virtual | Observational | Treatment | Specialist | Face, content and construct validity of the simulator | Intervention showed face, content and construct validity. |
| Milner et al. Eur Arch Otorhinolaryngol 2020. [34] | Brain | Simulation: ovine vs porcine model | Virtual | Observational | Treatment | Specialist | Face, global content and task specific content validity | Ovine model was statistically superior to the porcine model across most validation criteria, excluding realistic appearance of skin, identification and ligation of the trans-oral parotid duct and facial nerve grafting. |
| Bairamian et al. Neurosurgery 2019. [33] | Brain | VR angiogram vs 3D printed angiogram | Virtual | Observational | Treatment | Specialist | Face, content and construct validity and practicality | The VR angiogram was better for resolution; the 3D model was better for depth perception and manipulation. |
| Herder et al. Mil Med 2019. [27] | Brain | Swine vs synthetic model for lateral canthotomy | Virtual | Observational | Treatment | Specialist and medical student | Post intervention score | The synthetic trainer was easier to use. All groups improved their post intervention scores. |
| Yu et al. World Neurosurg 2019. [29] | Spinal Cord | Mixed Reality (MR) for pre-op planning | Virtual | Observational | Treatment | Specialist | Participant Feedback | Subjects found the intervention to be beneficial to training. Reduced puncture times and fluoroscopy times were noted. |
| Breimer et al. Oper Neurosurg 2017. [32] | Brain | Endoscopic third ventriculostomy (ETV) simulator | Virtual | Observational | Treatment | Specialist | Participant feedback | VR is helpful for learning anatomy and decision-making, while the physical simulator aided manual dexterity and technical skills. |
| Gasco et al. World Neurosurg 2013. [28] | Brain | Haemostasis simulator | Virtual | Observational | Treatment | Specialist and medical student | Participant  Feedback | Participants deemed the intervention suitable for training and important for developing career interest in medical students. |

**Supplementary Table 2.** Studies exclusively assessing by learning assessment (K&K Level 2).

| Study (Author/Year) | Disease Area | Intervention | Method  (virtual vs non virtual) | Type of study | Objective of intervention | Audience | Main outcome (s) | Findings |
| --- | --- | --- | --- | --- | --- | --- | --- | --- |
| Nasciento et al. Epileptic Disord. 2024;26(4):444-459. | Brain | Epileptiform diagnosis task feedback via web or phone application vs control | Virtual | Randomized controlled trial (RCT) | Diagnosis | Specialists | Knowledge | Intervention groups > control. |
| Zhang et al. BMC Med Educ. 2024. | All | Flipped classroom vs control | Non-virtual | RCT | Diagnosis | Specialists | Knowledge | Intervention group > control. |
| Yohannan et al. BMC Med Educ. 2024. | Brain | 3 Dimensional (3D) teaching (stereoscopic group / monoscopic group) vs control for neuroanatomy | Virtual | RCT | Diagnosis | Medical students | Knowledge | Both interventions groups > control. |
| Yilmaz et al. J Surg Educ. 2024. | Brain | Type of feedback (4 types) vs control. . | Virtual | RCT | Treatment | Medical students | Knowledge | Intervention groups > control. |
| Pitts et al. Am J Speech Lang Pathol. 2023. | All | Cadaver group vs control group | Non-virtual | Observational | Diagnosis | Medical Student | Knowledge | Intervention group > control. |
| Mohammadi et al. Iran J Pediatr. 2023. [38] | Brain | In person vs. virtual electroencephalogram (EEG) training. | Both | Observational | Diagnosis | Specialists | Knowledge | Both groups improved post intervention with no significant difference between groups. |
| Philbrick et al. World neurosurg 2023. [39] | Brain | Identifying Kocher’s point with navigation vs control | Virtual | RCT | Treatment | Medical students | Time and accuracy of identification | Intervention > control |
| Kiyofuji et al. Oper Neurosurg 2023. [40] | Brain | Slides vs simulator (intervention) | Virtual | RCT | Treatment | Specialists | Objective measure score | No significant difference between groups |
| Na et al. Anat sci educ 2022.[41] | Brain | Art training vs control | Non-virtual | RCT | Diagnosis | Medical students | Knowledge | Both groups improved |
| Mei-ling et al. Nurse educ today 2022. [42] | Brain | VR simulator vs lecture (control) | Virtual | Observational | Diagnosis and treatment | Allied professionals | Knowledge | Intervention > control |
| Shah et al. J Ultrasound Med 2021. [43] | All | Ultrasound participants vs control | Non-virtual | Observational | Examination | Medical students | Knowledge score | No difference in performance |
| Bornkamm et al. Neurology 2021. [44] | All | Blended teaching vs traditional teaching | Virtual | Observational | Examination | Medical students | Knowledge score | Intervention group > control |
| Greuter et al. Neurosurg focus 2021. [45] | Brain | 3D VR vs control | Virtual | RCT | Diagnosis | Specialists and Medical students | Time to aneurysm detection | Aneurysm detection time better in intervention arm |
| Wang et al World Neurosurg 2020.[46] | Brain | Microsurgical space restrictor vs control | Virtual | RCT | Treatment | Specialists | Objective post intervention test | Intervention > control |
| Ros et al. neurochirugie 2020. [47] | Brain | Technical note + VR simulation vs control | Virtual | RCT | Treatment | Medical students | Knowledge score | Intervention group > control |
| Argalious et al. J Cardiothorac Vasc Anesth 2019. [35] | Spinal Cord | Simulation vs problem-based learning (PBL) | Virtual | RCT | Treatment | Specialists | Composite score | No significant difference between groups |
| Meurer et al. otol neurotol 2019. [48] | Brain | Narrated slides and vignettes vs control | Non-virtual | RCT | Diagnosis | Specialists | Knowledge | Intervention group > control |
| Shi et al. World Neurosurg 2018. [36] | Spinal Cord | Virtual surgery vs cadaver (control) | Virtual | RCT | Treatment | Specialists | Screw penetration rate | Intervention group > control |
| Ciechanski et al. World Neurosurg 2017. [49] | Brain | Transcranial direct stimulation vs control | Virtual | RCT | Treatment | Medical students | Change in amount of Tumour resected post intervention | Intervention group > control |
| Arantes et al. Anat Sci Educ 2017. [18] | All | Course vs control (non-attendance) | Non-virtual | observational | Diagnosis | Non-specialists | Knowledge | Intervention arm > control |
| Clarke et al. BMC Medical Educ 2016. [50] | Brain | Instrument trainer + simulator vs control | Virtual | RCT | Treatment | Specialists | Total score | Intervention group > control |
| Belykh at al. J Neurosurg 2016. [51] | Brain | Human placental vessels vs bovine placental vessels | Non-virtual | Observational | Treatment | Specialists and medical students | Anastomosis assessment score | Both beneficial models with face, content, construct validity |
| Thawani et al. J Clin Neurosci 2016. [52] | Brain | Simulator vs control | Virtual | observational | Treatment | Specialists | Performance score | Intervention > control |
| Shah et al. Oper Neurosurg 2016.[53] | Brain | 3D simulator vs lecture (control) | Virtual | Observational study | Diagnosis | Specialists | Knowledge | Intervention > control |
| Kockro et al. Ann Anat 2015. [54] | Brain | Lecture + 3D intervention vs control | Virtual | RCT | Diagnosis | Medical students | Knowledge | Intervention non-inferior to control. |
| Patel et al. Neurol Res 2014. [55] | Brain | Simulator vs control | Virtual | observational | Treatment | Medical students | Knowledge score for identifying structures | Intervention > control |
| Johnson et al. Med Teach 2013. [56] | Brain | Group learning vs. individual (control) | Non-virtual | RCT | Diagnosis | Medical students | Knowledge | Intervention > control |
| Park et al. Med Teacher 2011. [57] | All | No feedback vs written only vs written and verbal | Non-virtual | RCT | Examination | Medical students | Knowledge | Written and verbal feedback had the greatest scores. |
| Schuh et al. J Clin Neurophysiol 2008. [58] | All | Weekly presentations + game vs didactic (control) | Non-virtual | observational | Examination | Medical students | Knowledge score for neurophysiology and overall USMLE | Intervention group > in neurophysiology. No difference in USMLE. |
| Ochoa et al. Teach Learn Med 2008. [59] | Brain | Web- based teaching vs traditional (control) | Virtual | RCT | Diagnosis | Medical students | Knowledge score | Intervention group > control |
| Levinson et al. Med Education 2007. [60] | Brain | Learner control vs programme control | Virtual | RCT | Diagnosis | Medical students | Knowledge | Programme control group attained the best score. |
| Schuh et al. Neurologist 2007. [37] | All | Weekly team oral/ written quiz intervention vs. control | Non-virtual | observational | Examination | Specialists | Knowledge score | Team quizzing improved scores the most. |

**Supplementary Table 3** Studies assessing educational interventions by behavioural changes (K&K level 3) or a combination of K&K Levels.

| Study (Author/Year) | Disease Area | Intervention | Method  (virtual vs non virtual) | Type of study | Objective of intervention | Audience | Main outcome (s) | K&K level | Findings |
| --- | --- | --- | --- | --- | --- | --- | --- | --- | --- |
| K&K Level 3 | | | | | | | | | |
| Saposnik et al. MDM Policy Pract 2019[62] | Brain | Simulated reflection-based intervention vs control | Non-virtual | Randomized controlled trial (RCT) | Treatment | Specialist | Reduction in therapeutic inertia | 3 | Intervention > control. |
| Sundar et al. J Neurosurg 2016.[61] | Spinal Cord | Simulation with navigation + cadaveric models vs control | Virtual | Observational | Treatment | Specialist | Surgical error | 3 | Intervention > control |
| K&K Combination | | | | | | | | | |
| Koka et al. J Med Internet Res. 2025. | Brain | E-learning group vs Video group | Virtual | RCT | Diagnosis and Treatment | Medical students and Allied Health Professionals (AHPs) | Knowledge and satisfaction | 1 and 2 | Both groups improved; however, intervention group > comparator group. |
| Xuan et al. BMC Med Educ. 2024. | Brain | Database learning vs control. | Virtual | Observational | Diagnosis | Medical Students | Knowledge and satisfaction | 1 and 2 | Intervention > control. |
| Li et al. BMC Med Educ. 2024. | All | Online course vs control group. | Virtual | RCT | Diagnosis and Treatment | Medical students | Knowledge and satisfaction | 1 and 2 | Intervention > control. |
| Toro J, et al.ÊBMC Res Notes. 2023;16(1):307. [68] | All | Didactic teaching vs virtual | Virtual and non-virtual | Observational | Examination | Medical students | Knowledge and satisfaction | 1 and 2 | No clinically significant difference in test results. |
| Robertson et al. J Neurosurg. 2023. [69] | Brain | Simulation + didactic vs simulation alone (control group) | Virtual | Observational | Treatment | Specialists | Scores on objective assessment of neurosurgical skill | 2 and 3 | Intervention group had improved technical skills and those early in their career showed a change in behaviour |
| Tarolli et al. Neurology 2023. [70] | All | Team based learning intervention. Pre- vs Post- intervention scores. | Non-virtual | Observational | Diagnosis | Medical students | Student opinion | 1 and 2 | Non-inferior results in the post-intervention group |
| Fazlollahi et al. JAMA netw open 2022. [71] | Brain | Feedback from either a Virtual Operator Assistant, VOA (VOA group) or a remote instructor (instructor group) vs no feedback (control) | Virtual | RCT | Treatment | Medical students | Procedural score and blinded Objective Structured Assessment of Technical Skills (OSATS) for VR tumour resection | 1 and 2 | VOA group > instructor and control group. |
| Rajan et al. BMC Med Educ 2022. [72] | Brain | Interactive e-module vs Wikipedia page without interaction (control) | Virtual | RCT | Diagnosis and treatment | Medical students | Knowledge, enjoyability, engagement and usefulness | 1 and 2 | Intervention was more engaging, useful and enjoyable.  No difference in quiz scores between the two groups. |
| Jiang et al. Medicine 2022. [73] | Brain | 3D printing + PBL vs traditional teaching (control) | Virtual | Observational | Diagnosis | Medical students | Knowledge and clinical practice skills | 1 and 2 | Intervention > control, in terms of scores, satisfaction, learning interest and spatial thinking ability. |
| Shen et al. BMC Med Educ 2022. [74] | All | CBL-PBL vs traditional teaching (control) | Non-virtual | RCT | Diagnosis | Medical students | Satisfaction and knowledge and self-assessed competence | 1 and 2 and 3 | Intervention > control. Members of the intervention group were more interested  and more often recruited to neurosurgery. |
| Kong et al. Ann Palliat Med. 2021. [75] | All | Microlecture vs control | Virtual | RCT | Diagnosis | Medical students | Teaching effect, theoretical operation, and clinical practice satisfaction | 1 and 2 | Intervention > control |
| Perin et al. World Neurosurg 2021. [64] | Brain | Pre-op planning using 3D navigation CT-MADE vs 2D traditional neuro-navigation for surgical planning (control) | Virtual | Observational | Treatment | Specialists | Surgical procedure and patient outcome | 1 and 4 | No difference between the 2 groups in procedure duration or post-op visual impairment.  Simulated craniotomies were significantly smaller than the real ones. The simulator helped residents improve their anatomic and procedural comprehension and was deemed a useful aid to ensure safety. |
| Rezende et al. Anat Sci Educ 2020. [76] | All | Team based learning vs traditional lectures (control) | Non-virtual | Observational | Diagnosis | Medical students | Knowledge acquisition and perception | 1 and 2 | Knowledge acquisition was similar between TBL and traditional lectures. TBL was associated with greater acceptance, higher motivation and better student perception. |
| Roth et al. J Grad Med Educ 2020. [77] | All | Written modules vs podcasts | Virtual | RCT | Diagnosis and treatment | Specialists | Knowledge and satisfaction | 1 and 2 | All groups improved post-intervention Podcast group had higher satisfaction. |
| Sun et al. World Neurosurg 2018. [66] | Spinal Cord | Problem-based simulation learning vs traditional teaching (control) in performing a lumbar puncture (LP) | Virtual | Observational | Treatment | Specialists | Knowledge scores.  Extent that residents are ready to perform LP. Successful LPs. | 1 and 2 | No difference in knowledge scores. Intervention > control at readiness and success in performing LP. |
| Stepan et al. Int Forum Allergy Rhinol 2017. [78] | Brain | VR interactive model vs online textbook (control) | Virtual | RCT | Diagnosis | Medical students | Knowledge | 1 and 2 | No difference in anatomy knowledge between the groups. The VR group found the learning experience to be significantly more engaging, enjoyable and useful. |
| Boody et al. Global Spine J 2017. [67] | Spinal Cord | 40-minute bio-skills training module vs self-directed study (control) | Virtual | Observational | Treatment | Medical students and Specialists | OSATS and self reported score. | 1 and 2 | Intervention > control. |
| Rampinelli et al. World Neurosurg 2017. [79] | Brain | 2D vs 3D in the endoscopy | Virtual | Observational | Treatment | Specialists | Time needed to complete each task | 1 and 2 | 3D technology conferred an advantage in terms of time of execution and user comfort. |
| Moisi et al. World Neurosurg 2017.[63] | Spinal Cord | Microscope vs exoscope (extracorporeal video microscope) | Virtual | Observational | Treatment | Specialists | Time of procedure, grading of decompression, complications | 1 and 4 | No difference in procedure time or complications. Greater comfort with exoscope. |
| Hsu et al. Nurse Educ today 2016. [80], [81] | All | Outcome based course design vs control | Non-Virtual | RCT | Treatment | Allied professionals | Nursing competency and satisfaction | 1 and 2 | No statistically significant difference in nursing competency score between groups. Satisfaction higher in intervention group. |
| Yang et al. BMC Med Educ 2014. [82] | All | Group A: Team based learning (TBL) + Lecture based learning (LBL); Group B: LBL only; Group C: TBL only | Non-Virtual | RCT | Diagnosis and treatment | Medical students | Knowledge test and satisfaction | 1 and 2 | Groups A and B had similar scores, both of which were significantly higher than group C. Theoretical and total scores for group A were significantly higher than groups B and C. All participants in group A were satisfied with TBL + LBL. |
| Willett et al. J Allied Health 2008. [81] | All | Computer based instruction (CBI) vs lecture-based teaching (control) | Non-Virtual | RCT | Treatment | Allied professionals | Exam scores, study time and student opinions | 1 and 2 | No differences in exam scores between groups. AHPs in CBI group spent less time studying. No major preference between students. |
| Heckmann et al. Eur J Neurol 2008. [83] | All | Peer taught vs experienced teachers (control) | Non-Virtual | RCT | Examination | Medical students | Knowledge | 1 and 2 | No difference between groups. Self-assessed learning success was rated equally. |
| Holloway et al Ann intern med 1999. [65] | Brain | Combined education strategy in dementia care vs control group | Non-Virtual | RCT | Treatment | Specialists | Adherence to recommendations, based on survey + patient chart review | 3 and 4 | Intervention group >control group. |

**Supplementary Table 4**. Newcastle Ottawa Scale risk of bias assessment

| Study ID | Selection | | | | Comparability (2) | Outcome | | | Total (9) |
| --- | --- | --- | --- | --- | --- | --- | --- | --- | --- |
|  | **Representativeness of Cohort (1)** | **Selection of non-exposed cohort (1)** | **Ascertainment of exposure (1)** | **Outcome present at start of study (1)** |  | **Assessment of outcome (1)** | **Follow up length (1)** | **Adequacy of follow up (1)** |  |
| Damon Et al. Operative Neurosurgery 2023 | 1 | 1 | 1 | 1 | - | - | 1 | 1 | 6 |
| Toro J, et al. ÊBMC Res Notes. 2023. | 1 | 1 | 1 | 1 | 1 | - | 1 | 1 | 7 |
| Robertson et al. J Neurosurg. 2023 | 1 | 1 | 1 | 1 | - | 1 | 1 | 1 | 7 |
| Mohammadi et al. Iran J Pediatr. 2023 | 1 | 1 | 1 | 1 | - | - | 1 | 1 | 6 |
| Tarolli et al. Neurology 2023 | 1 | 1 | 1 | 1 | - | - | 1 | - | 5 |
| Bornkamm et al. Neurology 2021. | 1 | 1 | 1 | 1 | - | - | 1 | 1 | 6 |
| Jiang et al. Medicine 2022. | 1 | 1 | 1 | 1 | - | 1 | 1 | 1 | 7 |
| Mei-ling et al. Nurse educ today 2022. | 1 | 1 | 1 | 1 | - | - | 1 | - | 5 |
| Shah et al. Oper Neurosurg 2016. | 1 | 1 | 1 | 1 | - | 1 | 1 | - | 6 |
| Perin et al. World Neurosurg 2021. | 1 | 1 | 1 | - | - | - | - | - | 3 |
| Ledwos et al Oper Neurosurg 2020. | 1 | 1 | 1 | 1 | 1 | 1 | 1 | - | 7 |
| Rezende et al. Anat Sci Educ 2020. | 1 | 1 | 1 | 1 | 1 | - | 1 | 1 | 7 |
| Milner et al. Eur Arch Otorhinolaryngol 2020. | 1 | 1 | - | 1 | - | - | 1 | 1 | 5 |
| Bairamian et al. Neurosurgery 2019. | 1 | 1 | - | 1 | - | - | 1 | 1 | 5 |
| Herder et al. Mil Med 2019. | 1 | 1 | 1 | 1 | - | - | 1 | - | 5 |
| Yu et al. World Neurosurg 2019. | 1 | 1 | 1 | 1 | - | - | 1 | - | 5 |
| Breimer et al. Oper Neurosurg 2017. | 1 | 1 | 1 | 1 | - | - | 1 | - | 5 |
| Boody et al. Global Spine J 2017. | 1 | 1 | 1 | 1 | 1 | 1 | 1 | 1 | 8 |
| Rampinelli et al. World Neurosurg | 1 | 1 | 1 | 1 | - | - | 1 | 1 | 6 |
| Moisi et al. World Neurosurg 2017. | 1 | 1 | 1 | 1 | - | 1 | 1 | - | 6 |
| Arantes et al. Anat Sci Educ 2017. | 1 | 1 | 1 | 1 | 1 | 1 | 1 | 1 | 8 |
| Belykh at al. J Neurosurg 2016. | 1 | 1 | 1 | 1 | - | 1 | - | - | 5 |
| Sundar et al. J Neurosurg 2016 | 1 | 1 | 1 | 1 | 1 | 1 | 1 | 1 | 8 |
| Thawani et al. J Clin Neurosci 2016. | 1 | 1 | 1 | 1 | - | 1 | 1 | 1 | 7 |
| Patel et al. Neurol Res 2014 | 1 | 1 | 1 | 1 | - | 1 | - | 1 | 6 |
| Schuh et al. J Clin Neurophysiol 2008. | 1 | 1 | 1 | 1 | 1 | 1 | 1 | - | 7 |
| Schuh et al. Neurologist 2007. | 1 | 1 | 1 | 1 | 1 | 1 | 1 | - | 7 |
| Sun et al. World Neurosurg 2018. | 1 | 1 | 1 | 1 | - | 1 | 1 | - | 6 |
| Shah et al. J Ultrasound Med 2021. | 1 | 1 | 1 | 1 | - | 1 | 1 | - | 6 |
| Gasco et al. World Neurosurg 2013. | 1 | 1 | 1 | 1 | - | - | 1 | - | 5 |
| Pitts et al. Am J Speech Lang Pathol. 2023. | 1 | 1 | 1 | 1 | 1 | 1 | 1 | 1 | 8 |
|  |  |  |  |  |  |  |  |  |  |
|  |  |  |  |  |  |  |  |  |  |


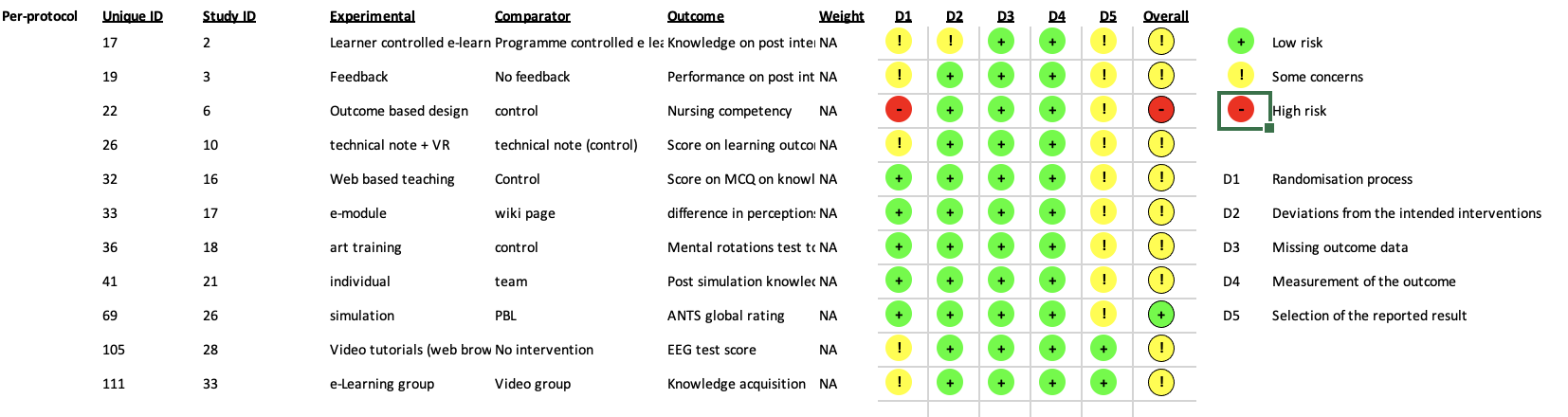

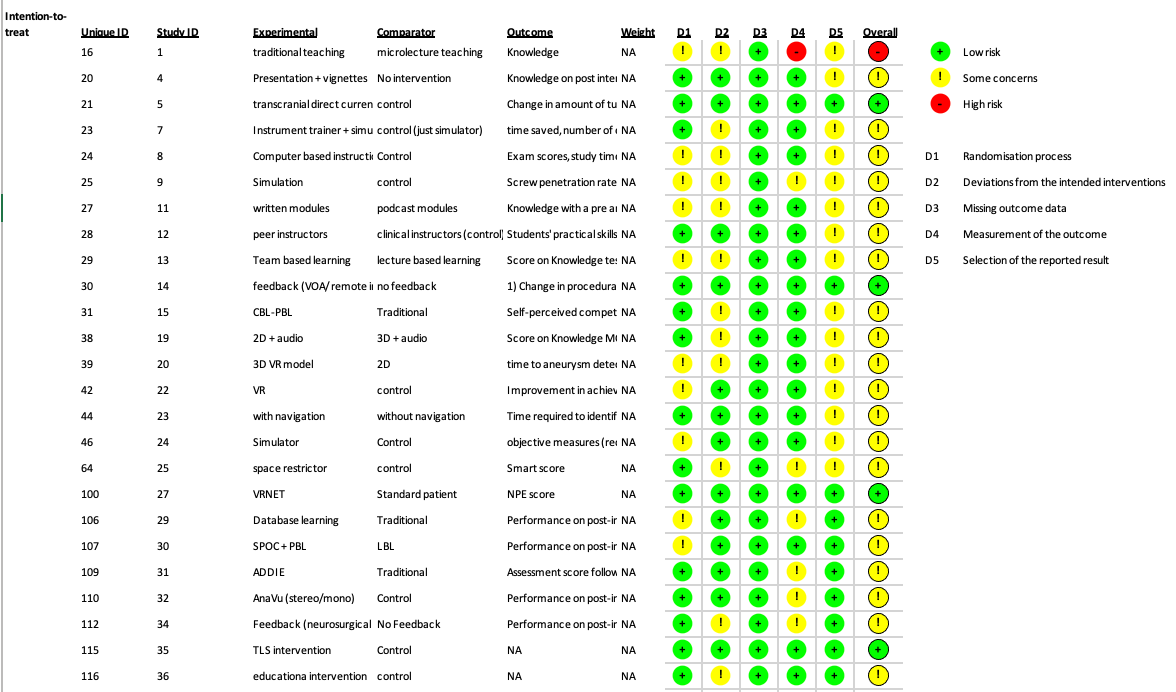


**Supplementary Figure 3**. Cochrane risk of bias assessment for Randomized Controlled Trials

**Supplementary Figure 4**. Funnel plot of Randomized Controlled Trials in Forest plot.


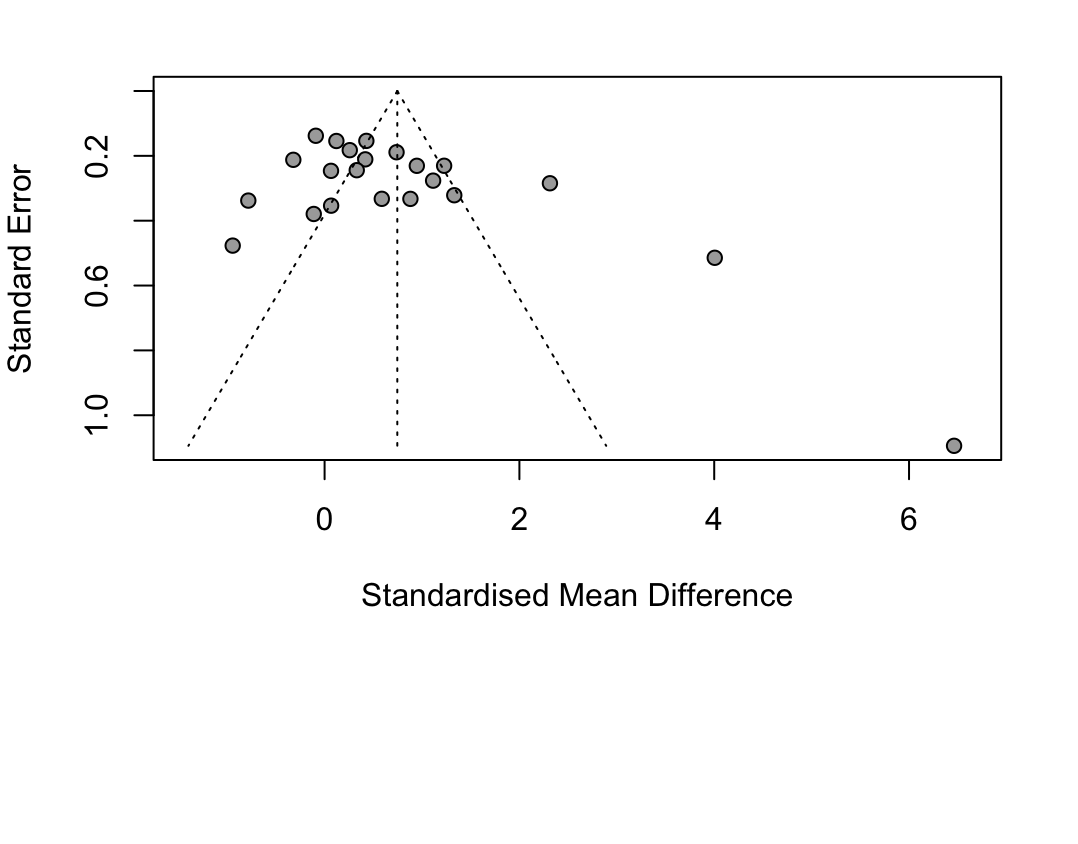

Supplement: online supplemental file 1 [file bmjopen-15-11-s001.docx]
