# Supplementary material for: Comparative effectiveness of educational interventions in neurological disease for healthcare workers and students: a systematic review
Source: BMJ Open. 2025 Nov 27;15(11):e107475. doi: 10.1136/bmjopen-2025-107475 (PMC12666186; doi:10.1136/bmjopen-2025-107475)
Supplement: Supplementary file 2 [file bmjopen-2025-107475.reviewer_comments.pdf]

PEER REVIEW HISTORY

BMJ Open publishes all reviews undertaken for accepted manuscripts. Reviewers are asked to complete a checklist review form and are provided with free text boxes to elaborate on their assessment. These free text comments are reproduced below.

ARTICLE DETAILS

Title (Provisional)

Comparative effectiveness of educational interventions in neurological disease for healthcare workers and students: a systematic review.

Authors

Veremu, Munashe; Jiang, Zhilin; Gillespie, Conor S; Roman, Elena; Cook, William H; Chauhan, Rohil; Fard, Amir Rafati; Toumbas, Georgios; Baig, Shehla; Zipser, Carl; Stacpoole, Sybil; Tetreault, Lindsay; Deakin, Naomi; Bateman, Antony; Davies, Benjamin

VERSION 1 - REVIEW

**Reviewer**	**1**
**Name**	**Saposnik, Gustavo**
**Affiliation**	**University of Toronto**
**Date**	**07-Aug-2025**
**COI**	**None**

Reviewer Comments on Manuscript

I had the opportunity to review the present manuscript. Below are my observations and suggestions for improvement.

Strengths

1. The topic is timely and relevant, addressing an important gap in medical education.

2. The authors have attempted to synthesize a body of literature on educational interventions related to neurology.

General Considerations and Limitations

1. Introduction – Neurophobia Rationale

The rationale invoking neurophobia is underdeveloped. While neurophobia is a recognized phenomenon, the importance of medical education in neurology transcends students' perceived limitations. I recommend strengthening the rationale by connecting neurophobia to broader educational challenges and clinical outcomes.

2. Literature Search – Incomplete Scope

The literature search appears limited. When replicating the methodology, I identified additional relevant studies not included in the manuscript. I suggest expanding the search and incorporating these studies to enhance the comprehensiveness of the review. A few examples are listed below for reference.

Randomized trial on the impact of card Game-Based teaching on learning and memory retention of neurological syndromes.

Yu X, Wu J, Zhang Y, Di Z, Nie W, Wang M, Zhu X, Zhang Y, Wu Y, Ma Y, Han Y, Yu M.

BMC Med Educ. 2025 Jul 13;25(1):1046. doi: 10.1186/s12909-025-07630-9.

PMID: 40653496 Free PMC article. Clinical Trial.

Evaluation of a flipped classroom approach to undergraduate neurology medical education in Zambia.

Holroyd KB, McDonough A, Chomba M, Asukile M, Chiwaya N, Vallejos G, Asmare S, Braun S, Chishimba L, Gebrewold MA, Luchembe M, Mulenga B, Mwamba J, Namangala C, Nassoro D, Peloso A, Yankae L, Zimba S, Berkowitz A, Saylor D.

J Neurol Sci. 2025 Aug 15;475:123580. doi: 10.1016/j.jns.2025.123580. Epub 2025 Jun 11.

PMID: 40543399 Clinical Trial.

Evaluation of prescribing patterns of sodium valproate in neurological disease patients: a quasi-experimental pretest-posttest design study.

Werida RH, El-Sherif S, Shoshan R, Khedr NF.

BMC Health Serv Res. 2025 Mar 27;25(1):441. doi: 10.1186/s12913-025-12483-5.

PMID: 40140829 Free PMC article. Clinical Trial.

Virtual reality-based neurological examination teaching tool(VRNET) versus standardized patient in teaching neurological examinations for the medical students: a randomized, single-blind study. Han SG, Kim YD, Kong TY, Cho J. BMC Med Educ. 2021 Sep 15;21(1):493. doi: 10.1186/s12909-021-02920-4. PMID: 34526004 Free PMC article. Clinical Trial.

Traffic Lights Intervention Reduces Therapeutic Inertia: A Randomized Controlled Trial in Multiple Sclerosis Care.

Saposnik G, Mamdani M, Montalban X, Terzaghi M, Silva B, Saladino ML, Tobler PN, Caceres F. MDM Policy Pract. 2019 Jun 21;4(1):2381468319855642. doi: 10.1177/2381468319855642. eCollection 2019 Jan-Jun. PMID: 31259250

Holloway RG, Gifford DR, Frankel MR, Vickrey BG. A randomized trial to implement practice recommendations: design and methods of the Dementia Care Study. Control Clin Trials. 1999 Aug;20(4):369-85. doi: 10.1016/s0197-2456(99)00006-9. PMID: 10440564.

There are more....

3. Meta-Analysis – Missing Quantitative Synthesis

Although the authors explain why a meta-analysis was not conducted, they do not clarify why efficacy estimates from randomized trials could not be extracted. Including even a basic pooled effect size or standardized mean difference (SMD) would strengthen the manuscript's impact.

4. Results – Clarity and Structure

The results section is difficult to follow. The tables primarily describe methodological aspects but do not clearly convey the efficacy of educational interventions. Consider reorganizing the tables to highlight outcomes and comparative effectiveness, especially given the study's main objective.

5. Comparative Effectiveness – Critical Gap

This section is central to the manuscript but lacks clarity. Effectiveness rates are reported without comparative success metrics between intervention and control groups. For cohort studies, the nature of the intervention should be summarized in the same table to avoid forcing readers to cross-reference multiple sections.

6. Abstract – Interpretation of Outcomes

The abstract states that 69% of studies favored the intervention, but this does not convey the magnitude of benefit. Consider reporting mean effect sizes or SMDs to help readers understand the likely treatment effect, especially since randomized trials are included.

7. Discussion – Length vs. Depth

The discussion spans approximately six pages (~1800 words) but lacks synthesis of key findings. I recommend condensing this section while emphasizing the implications and limitations of the results.

8. Language and Reliability

The manuscript would benefit from thorough proofreading to improve clarity, grammar, and consistency. Optimizing the language will enhance the reliability and professionalism of the work.

I hope the authors find these comments constructive and helpful in strengthening the manuscript.

**Reviewer**	**2**
**Name**	**Frey , Jessica**
**Affiliation**	**West Virginia University, Neurology**
**Date**	**25-Aug-2025**
**COI**	**None**

This is a nice overview of all the different educational intervention studies in the neurology field. While the study is limited by the variability and heterogeneity of the studies the authors do a nice job summarizing the existing studies and the tables have a lot of valuable information. However, I do have some minor and major comments for the authors to consider:

Minor Comments:

Line 85- “clinical the impact” please revise.

Line 115: what is “NICE”?

Major Comments:

Introduction

Seems to go on for a long time. Unclear if the main concern is that lack of neurologic knowledge leads to poor patient outcomes or rather that there is not enough education on neurologic disease? If the argument is that lack of neurologic disease leads to poor outcomes, what is the evidence for that? Has that been formally studied? Beyond the example of cervical myelopathy, I do not actually see much evidence that poor knowledge leads to poor outcomes, only that it leads to delayed referral or delayed diagnosis.

In reading on to your results section, demonstrating that there were 0 studies that assess Kirkpatrick 4 according to Table 2, would consider substantial revision to the introduction. A refocus away from education = improvement in clinical results since that cannot be demonstrated by available data.

Also do we know WHY we have lack of knowledge? If this due to poor education techniques (since the review is trying to demonstrate what educational techniques might be useful)? Or are there other reasons for the lack of knowledge, such as lack of exposure? Do trainees have mandatory neurology during their training? A background on what the typical training for your target population is will be helpful for the reader to understand how an educational intervention might be helpful for mitigating the challenges you have described in the introduction.

Methods

How do you define healthcare professionals? Medical students, residents, faculty? Nurses, physician assistants, nurse practitioners?

Some educational interventions are used purely to garner interest from a population of learners, and not to actually change knowledge. Did your criteria exclude studies that were purely to excite learners or to introduce learners to a new topic but not necessarily change their knowledge base?

Primary outcome measure of “effectiveness” is vague. How do you define this? Was this defined differently in each study? If so, this should be reported as a limitation as well.

Results

The tables are very nicely done, separated out by K&K criteria. It may be helpful to have additional detail provided in the supporting narrative paragraphs. For example for K&K level 1 paragraph, can you indicate what the interventions actually consisted of beyond “simulation.” Were these one-time events, were they longitudinal, were the lecture-based, or procedure-based? I am guessing there will be quite a bit of heterogeneity amongst the actual intervention.

When you use “specialist” how are you defining this? Is this a neurology specialist? Non-neurology specialist?

For K&K level 2, can you define how knowledge was measured for each study? Were these validated knowledge-based scales? Standardized tests? Or quizzes that were produced specifically for the purposes of that research study? Again, might be nice to describe some of the most common styles of intervention in the narrative portion. For example, how many were longitudinal, one-time events, how many were lecture-based versus hands-on experiences, did any use patients?

For K&K with combination, again a brief summary of what the studies actually did beyond just that they were observational might be helpful to give some context to the reader.

It might be helpful to add subheadings to help organize the narrative portion of the paragraphs, and to always place in the same order, as it feels a little confusing to read as it currently stands. For example, in K&K combined, the authors reflect on:

-observational vs RCT

-virtual or non-virtual

-generic content focus (treatment, diagnosis, etc)

-target population

-neurologic localization

It looks like the other K&K paragraphs are somewhat organized in this matter, but this is not clear at first glance, and I had trouble navigating these paragraphs. Subheadings may help. I would also consider adding a portion that actually talks about what the studies did (were these lectures? Hands-on experiences? Longitudinal? One time experiences? Etc). While the table does a nice job with the details, adding in an overview of the style of the educational experience would be helpful for the narrative portion too.

Discussion

The discussion may also need to be revised depending on how you reframe the introduction. While the authors nicely acknowledge the heterogeneity of the studies, it does make it difficult to draw any overarching conclusions. Thus the key findings paragraph may need to be revised to reflect the updated outcomes/goals that you will list in the introduction section (see my prior comment from above).

For future directions, I recommend adding some specific guidelines for authors to consider as they create their own educational studies in the future. Should they only be RCTs? Should they aim to measure all 4 of the K&K levels? Should they use validated testing measures for measurements of knowledge? Etc.

VERSION 1 - AUTHOR RESPONSE

Reviewer 1:

Reviewer Comments on Manuscript

I had the opportunity to review the present manuscript. Below are my observations and suggestions for improvement.

Strengths

1. The topic is timely and relevant, addressing an important gap in medical education.
2. The authors have attempted to synthesize a body of literature on educational interventions related to neurology.

- Thank you for your kind comments.

General Considerations and Limitations

1. Introduction – Neurophobia Rationale

The rationale invoking neurophobia is underdeveloped. While neurophobia is a recognized phenomenon, the importance of medical education in neurology transcends students' perceived limitations. I recommend strengthening the rationale by connecting neurophobia to broader educational challenges and clinical outcomes.

- *Thank you for this comment. We have provided further development of this in the 1st paragraph of the article to address the reviewers concerns.*

2. Literature Search – Incomplete Scope

The literature search appears limited. When replicating the methodology, I identified additional relevant studies not included in the manuscript. I suggest expanding the search and incorporating these studies to enhance the comprehensiveness of the review. A few examples are listed below for reference.

- *Thank you to the reviewer for this comment.. Our literature search was conducted systematically using predefined eligibility criteria. We acknowledge that additional relevant studies may now be available or may not have been captured by our search terms. Where possible, we have reviewed the examples suggested by the reviewer and noted their relevance. While we did not repeat the full search, acknowledged that our findings represent the evidence identified within the scope of our predefined strategy. We have responded in in turn with each paper suggested below.*

Randomized trial on the impact of card Game-Based teaching on learning and memory retention of neurological syndromes.

Yu X, Wu J, Zhang Y, Di Z, Nie W, Wang M, Zhu X, Zhang Y, Wu Y, Ma Y, Han Y, Yu M. BMC Med Educ. 2025 Jul 13;25(1):1046. doi: 10.1186/s12909-025-07630-9.

PMID: 40653496 Free PMC article. Clinical Trial.

Evaluation of a flipped classroom approach to undergraduate neurology medical education in Zambia.
Holroyd KB, McDonough A, Chomba M, Asukile M, Chiwaya N, Vallejos G, Asmare S, Braun S, Chishimba L, Gebrewold MA, Luchembe M, Mulenga B, Mwamba J, Namangala C, Nassoro D, Peloso A, Yankae L, Zimba S, Berkowitz A, Saylor D.
J Neurol Sci. 2025 Aug 15;475:123580. doi: 10.1016/j.jns.2025.123580. Epub 2025 Jun 11.
PMID: 40543399 Clinical Trial.

- Thank you for the suggestion of these articles. The above 2 articles were published after the search date and therefore would not be included.

Evaluation of prescribing patterns of sodium valproate in neurological disease patients: a quasi-experimental pretest-posttest design study.
Werida RH, El-Sherif S, Shoshan R, Khedr NF.
BMC Health Serv Res. 2025 Mar 27;25(1):441. doi: 10.1186/s12913-025-12483-5.
PMID: 40140829 Free PMC article. Clinical Trial.

- *This study would be excluded based on the eligibility criteria. The target population are patients and not healthcare professionals. All of the studies in our paper have the target population of healthcare professionals and not patients.*

Virtual reality-based neurological examination teaching tool(VRNET) versus standardized patient in teaching neurological examinations for the medical students: a randomized, single-blind study. Han SG, Kim YD, Kong TY, Cho J. BMC Med Educ. 2021 Sep 15;21(1):493. doi: 10.1186/s12909-021-02920-4.
PMID: 34526004 Free PMC article. Clinical Trial.

- *This study is already included.*

Traffic Lights Intervention Reduces Therapeutic Inertia: A Randomized Controlled Trial in Multiple Sclerosis Care.

Saposnik G, Mamdani M, Montalban X, Terzaghi M, Silva B, Saladino ML, Tobler PN, Caceres F. MDM Policy Pract. 2019 Jun 21;4(1):2381468319855642. doi: 10.1177/2381468319855642. eCollection 2019 Jan-Jun. PMID: 31259250

- *Thank you for highlighting this study, we have now included it.*

Holloway RG, Gifford DR, Frankel MR, Vickrey BG. A randomized trial to implement practice recommendations: design and methods of the Dementia Care Study. Control Clin Trials. 1999 Aug;20(4):369-85. doi: 10.1016/s0197-2456(99)00006-9. PMID: 10440564.

- Thank you for highlighting this study, this is a protocol paper for a trial, and although would potentially be included, protocol papers are excluded in the eligibility criteria.

We have found the RCT paper it refers to and have included it.

3. Meta-Analysis – Missing Quantitative Synthesis

Although the authors explain why a meta-analysis was not conducted, they do not clarify why efficacy estimates from randomized trials could not be extracted. Including even a basic pooled effect size or standardized mean difference (SMD) would strengthen the manuscript's impact.

- *Thank you to the reviewer for this comment. We have addressed this with calculation of a standardised mean difference for randomized controlled studies where it is calculable.*

4. Results – Clarity and Structure

The results section is difficult to follow. The tables primarily describe methodological aspects but do not clearly convey the efficacy of educational interventions. Consider reorganizing the tables to highlight outcomes and comparative effectiveness, especially given the study's main objective.

- We thank the reviewer for this comment. Although the tables do include methodological aspects, some of these aspects do form part of the secondary outcomes we were investigating from each study.

Therefore we feel they are relevant to be included in the summary table to give a wholistic picture of each study. We have organised each table in publication year order, with the most recent study coming up first.

5. Comparative Effectiveness – Critical Gap

This section is central to the manuscript but lacks clarity. Effectiveness rates are reported without comparative success metrics between intervention and control groups. For cohort studies, the nature of the intervention should be summarized in the same table to avoid forcing readers to cross-reference multiple sections.

- Thank you to the reviewer for this comment. We have included a section on the standardized mean differences within this section of the study, where we have pooled the standardized mean differences for the randomized controlled studies. Whilst we haven't edited the effectiveness table, we have added harvest plots to better visualise the comparative effectiveness and to show the distribution of effective studies between the different outcome domains (Kirkpatrick and Kirkpatrick model + different methodologies used).

6. Abstract – Interpretation of Outcomes

The abstract states that 69% of studies favored the intervention, but this does not convey the magnitude of benefit. Consider reporting mean effect sizes or SMDs to help readers understand the likely treatment effect, especially since randomized trials are included.

- Thank you to the reviewer for this comment. We have included the SMD as requested.

7. Discussion – Length vs. Depth

The discussion spans approximately six pages (~1800 words) but lacks synthesis of key findings. I recommend condensing this section while emphasizing the implications and limitations of the results.

- Thank you to the reviewer for this comment. We have condensed 700 words from the discussion.

8. Language and Reliability

The manuscript would benefit from thorough proofreading to improve clarity, grammar, and consistency. Optimizing the language will enhance the reliability and professionalism of the work.

- We thank the reviewer for this comment. We have taken a further look at the paper and have taken extra care to optimize the language.

Reviewer 2

- [ ] Minor Comments:Line 85- "clinical the impact" please revise.

Line 115: what is "NICE"?

Thank you for this comment. We have since revised line 85 and added a definition for NICE.

Major Comments:

- Introduction Seems to go on for a long time. Unclear if the main concern is that lack of neurologic knowledge leads to poor patient outcomes or rather that there is not enough education on neurologic

disease? If the argument is that lack of neurologic disease leads to poor outcomes, what is the evidence for that? Has that been formally studied? Beyond the example of cervical myelopathy, I do not actually see much evidence that poor knowledge leads to poor outcomes, only that it leads to delayed referral or delayed diagnosis. In reading on to your results section, demonstrating that there were 0 studies that assess Kirkpatrick 4 according to Table 2, would consider substantial revision to the introduction. A refocus away from education = improvement in clinical results since that cannot be demonstrated by available data. Also do we know WHY we have lack of knowledge? If this due to poor education techniques (since the review is trying to demonstrate what educational techniques might be useful)? Or are there other reasons for the lack of knowledge, such as lack of exposure? Do trainees have mandatory neurology during their training? A background on what the typical training for your target population is will be helpful for the reader to understand how an educational intervention might be helpful for mitigating the challenges you have described in the introduction.

- Thank you to the reviewer for this insightful comment. We agree that our review does not provide evidence that educational interventions directly improve patient outcomes (Kirkpatrick level 4), Our intent is to highlight that, for progressive neurological conditions, *early diagnosis is associated with improved clinical outcomes* — as shown in cervical myelopathy, and now expanded with additional examples including multiple sclerosis and ALS. The link we are emphasizing is that delayed or missed diagnoses often stem from limited neurological knowledge among healthcare professionals along the diagnostic pathway, which may contribute to worse outcomes. Our review further underlines this gap by demonstrating that very few studies evaluate the clinical outcomes of educational interventions at all.

Methods

How do you define healthcare professionals? Medical students, residents, faculty? Nurses, physician assistants, nurse practitioners?

- Thank you to the reviewer for this comment. In general, we are quite broad about our description of what a healthcare professional is; however, inherently this would likely be someone who is likely to encounter a patient with a neurological presentation which perhaps limits the the scope slightly. We have included further descriptions which professionals may be included.

Some educational interventions are used purely to garner interest from a population of learners, and not to actually change knowledge. Did your criteria exclude studies that were purely to excite learners or to introduce learners to a new topic but not necessarily change their knowledge base?

- Thank you to the reviewer for this comment. Part of our inclusion criteria was to include studies with the purpose of improving education of a neurological/neurosurgical disease(s). By this notion, if the

purpose of a study was to excite the participants, it would be excluded.

Primary outcome measure of "effectiveness" is vague. How do you define this? Was this defined differently in each study? If so, this should be reported as a limitation as well.

- Thank you to the reviewer for this comment. We have included a definition of effectiveness in the definitions section of the study. This is a based on whether the intervention results are better than the control.

Results

The tables are very nicely done, separated out by K&K criteria. It may be helpful to have additional detail provided in the supporting narrative paragraphs. For example for K&K level 1 paragraph, can you indicate what the interventions actually consisted of beyond "simulation." Were these one-time events, were they longitudinal, were the lecture-based, or procedure-based? I am guessing there will be quite a bit of heterogeneity amongst the actual intervention.

- We thank the reviewer for this comment. We have addressed this within the section that discussed K&K level 1.

When you use "specialist" how are you defining this? Is this a neurology specialist? Non-neurology specialist?

- Thank you to the reviewer for this comment. The term specialist is defined in the definitions section of the methods.

For K&K level 2, can you define how knowledge was measured for each study? Were these validated knowledge-based scales? Standardized tests? Or quizzes that were produced specifically for the purposes of that research study? Again, might be nice to describe some of the most common styles of intervention in the narrative portion. For example, how many were longitudinal, one-time events, how many were lecture-based versus hands-on experiences, did any use patients?

- Thank you to the reviewer for this comment. We have made some comments to address these comments.

For K&K with combination, again a brief summary of what the studies actually did beyond just that they were observational might be helpful to give some context to the reader. It might be helpful to add subheadings to help organize the narrative portion of the paragraphs, and to always place in the same order, as it feels a little confusing to read as it currently stands. For example, in K&K combined, the authors reflect on:

-observational vs RCT

-virtual or non-virtual

-generic content focus (treatment, diagnosis, etc)

-target population

-neurologic localization

It looks like the other K&K paragraphs are somewhat organized in this matter, but this is not clear at first glance, and I had trouble navigating these paragraphs. Subheadings may help. I would also consider adding a portion that actually talks about what the studies did (were these lectures? Hands-on experiences? Longitudinal? One time experiences? Etc). While the table does a nice job with the details, adding in an overview of the style of the educational experience would be helpful for the narrative portion too.

- Thank you to the reviewer for your comments. Whilst we have not added subheadings, we have added further narrative to the sections describing the different K&K models.

Discussion

The discussion may also need to be revised depending on how you reframe the introduction. While the authors nicely acknowledge the heterogeneity of the studies, it does make it difficult to draw any overarching conclusions. Thus the key findings paragraph may need to be revised to reflect the updated outcomes/goals that you will list in the introduction section (see my prior comment from above).

For future directions, I recommend adding some specific guidelines for authors to consider as they create their own educational studies in the future. Should they only be RCTs? Should they aim to measure.

- We thank the reviewer for this comment. Whilst we have not reframed the essence of the message of the article, we now report standardized mean differences (SMDs) from randomized studies where calculable, to illustrate the magnitude of effect across interventions. We acknowledge that heterogeneity continues to limit overarching conclusions and have highlighted this in the limitations. In the future directions section, we now provide more concrete guidance for study design — including the value of randomized controlled trials where feasible, the need for standardized and validated outcome measures, and the importance of moving beyond Kirkpatrick level 1–2 outcomes toward patient- and system-level endpoints when possible.

VERSION 2 - REVIEW

**Reviewer**	**2**
**Name**	**Frey , Jessica**
**Affiliation**	**West Virginia University, Neurology**
**Date**	**03-Nov-2025**
**COI**	

I believe the article is substantially improved with the revisions the authors have incorporated. I still feel that the heterogeneity amongst study time and participant population limits the ability of the authors to truly compare the studies but this has been acknowledged in the limitations section and I can see how an article collating this vast array of neuro-educational studies would still be valuable in spite of the associated limitations, so I do think it is worthwhile to move forward with publication.
